# Miscellaneous Notes

**Published:** 1872-06

**Authors:** 


### MISCELLANEOUS NOTES.

CARBOLIC ACID AS AN ANÆSTHETIC.—At the stated meeting of the Medical Society of the County of New-York, April 22, 1872, (*New-York Medical Journal*, June, 1872), Dr. Andrew H. Smith, from the Committee on Intelligence, read a Report on Therapeutics, from which we extract the following:

Dr. Bill, of the United States Army, and Dr. Squibb, have recently called attention to the action of carbolic acid as a local anæsthetic. As their papers have had a very general circulation, I will not give a synopsis of them, but merely state the results of some experiments that I have made upon my own person, which agree entirely with their experience.

In the first experiment, I painted a spot on the forearm, about an inch in diameter, with carbolic acid of about the strength of eighty-five per cent. For about a minute there was a slight burning sensation, after which the integument became entirely insensible, the cuticle being whitened and shriveled and the spot slightly elevated. I then with a scalpel made an incision about half an inch in length through the whole thickness of the integument. This was done without even feeling the contact of the knife. The capillary circulation seemed not to be materially interfered with, as the blood flowed as freely as it would from a similar wound under ordinary circumstances. The reparative process was also not impaired, adhesion taking place immediately. Three hours after the application of the acid, a needle could be thrust freely into the skin without causing pain.

In the second experiment, carbolic acid was applied as before, and ten minutes after, a fly-blister was placed upon the spot. The blister remained eight and a half hours without causing any pain, and without producing vesication.

In two instances, I have applied the acid previous to incising a whitlow. The operation was almost painless, but, as the whitlow was in each case of the superficial variety, the test was not entirely conclusive.

Inhaled in the form of spray, I have found the acid very useful in allaying irritation of the bronchial mucous membrane; coughs which have resisted all ordinary treatment have been immediately relieved, and in the course of two or three days, entirely removed.

I would suggest the use of a strong solution of carbolic acid as a revulsive, in cases in which a continuous impression is desired. While causing but little suffering, it produces an intense hyperæmia of the skin, which persists for eight or ten days, and is followed by desquamation of the cuticle.

New Observations on the Use of Arsenic.—(By L. Papilland. Condensed for the *Rundschau* by J. Lowry, from the *Gazette de Paris*. Translated by J. F. Noyes, M.D., Ophthalmic Surgeon to St. Mary's Hospital.)

Formerly arsenic was used only exceptionally as a last resort in skin diseases and fever. For the last fifteen years, however, the use of arsenic has greatly increased. Its effect upon nutrition, respiration and circulation is generally conceded. There are scarcely ten patients but that in eight of them, at least, it is indicated. Its full effect is attained only when it has been given many months, indeed a year, in quantities of .03 to .3 of a grain daily. It is especially efficacious in phthisis.

Pidaux, of Eaux Bonnes, to whom a very large number of patients with lung affections go, prescribes arsenic as the best remedy in phthisis. In the year 1869 he published a brochure upon phthisis, in which he concedes to arsenic the second rank as a therapeutic agent in this affection. The effect of arsenic upon the circulation was of late the subject of physiological and chemical experiments. Pidaux claims to have called attention to this subject in his articles on antimon-arsenii. He had shown that antimon-arsenii had a special effect upon the central organ of the circulation, and his idea has been confirmed by a great number of experiments, and, according to the observations of Pidaux, the arsenic must be taken for a long while consecutively. When once the improvement sets in, then the convalescence goes regularly forward. Cod-liver oil, quinia, raw flesh and alcohol improve the general system, in which also the lungs take a part, since the arsenic worked directly upon the diseased lungs. It gives to the diseased organ a power of resistance which the malady had limited.

For a long time arsenious acid has been given for intermittent fever, arsenite of soda for dyspepsia, and arsenite of soda and ammonia in skin diseases. Pidaux thinks, however, that the soluble compound should be used in diseases of shorter duration, as fever, neuralgia, icterus, and certain skin diseases; whereas the insoluble compounds should be given in diseases of longer duration, as heart affections, lung phthisis, bronchial catarrh, chloro-anæmia, dyspepsia, and in chronic skin diseases.—*Detroit Review of Medicine.*

Swinging in Phthisis.—The effect of swinging seems to have been observed long ago. George F. Elliott, M.D., states that in the year 1785, one Dr. Smith, F.R.S., tried the effect of swinging on fourteen consumptive patients, at the Middlesex Hospital. It was practiced twice a day, for half an hour at a time. On two of the patients, it seemed to have but little effect; in the remainder, the pulse fell from eight to fifteen beats per minute. His conclusion is, that "the motion of swinging has often a very sensible and immediate operation on the heart and lungs, as it reduces the frequency of the pulse, lessens febrile heat, suspends or prevents coughing, and promotes expectoration.—*New-York Medical Journal, June.*

Gelseminum in Irritable Bladder.—Dr. W. S. Hill, of Augusta, Maine, (*American Medical Journal*), reports five cases of irritable bladder cured by the fluid extract of gelseminum, of which ten to fifteen drops were given three or four times per day. In some cases, it was combined with bromide of potassium. The difficulty and pain in micturition were quite severe in two or three of the patients.—*Pacific Medical and Surgical Journal.*

Abortive Treatment of Erysipelas.—Dr. Piazza recommends, as a local means of effecting the cure of erysipelas, the application of a somewhat concentrated solution of silicate of potash, which should be painted in two or three successive layers on the part affected.—*Medical Record.*

Importance of Gonorrhœa in Producing Vesical Catarrh.—Dr. Kraus, of Vienna, in speaking of his recent experience in this subject, says that the best prophylactic against catarrh of the bladder is the employment of a scientific treatment of gonorrhœa, and he protests strongly against the introduction of irritating substances into the male urethra. It is just as difficult, he says, to cure vesical catarrh, when once established, as it is easy to cure gonorrhœa. Ricord, also, says that the great majority of catarrhs of the bladder are contracted from ill-treated gonorrhœas, the extension of the inflammation taking place in the acute stage of the latter affection. The last-mentioned writer affirms that he has not met with a case in which the vesical complication has taken place in patients whose gonorrhœas were treated by his method of treatment from the outset; but as many as eighty per cent. of the cases which came to him, after having been in the hands of charlatans, had suffered from the complication mentioned.

In the treatment of acute catarrh of the bladder, Dr. Kraus disapproves the use of diuretics, stimulants, and all the mineral waters which have been so fashionable, and directs the patient to drink a quantity of distilled water. This, although it increases the quantity of urine, renders it, by dilution, less irritating. The water should be taken neither hot nor cold, and may, if more agreeable to the patient, be flavored. Tepid baths are also of great service.—*The Medical Record, May,* 1872.

Removal of an Inverted Uterus.—Dr. Thomas Hay, of Philadelphia (*Medical and Surgical Reporter,* December, 1872), publishes a case in which an inverted uterus, with an intramural fibrous tumor of the fundus, was successfully removed by *écrasement* from a married woman, aged thirty-two years. The entire mass removed weighed one pound avoirdupois, less one quarter-ounce troy, and measured in length, 6½ inches; in circumference, at the thickest part, 12 inches; and around the neck, 8½ inches.—*The Medical Record, May,* 1872.

